# Melatonin Attenuates *β*-Glycerophosphate-Induced Calcification of Vascular Smooth Muscle Cells via a Wnt1/*β*-Catenin Signaling Pathway

**DOI:** 10.1155/2019/3139496

**Published:** 2019-12-11

**Authors:** Wei Ren Chen, Yu Jie Zhou, Jia Qi Yang, Fang Liu, Ying Xin Zhao, Yuan Sha

**Affiliations:** ^1^Department of Cardiology, Beijing Anzhen Hospital, Capital Medical University, Beijing Institute of Heart Lung and Blood Vessel Disease, Beijing Key Laboratory of Precision Medicine of Coronary Atherosclerotic Disease, Clinical Center for Coronary Heart Disease, Capital Medical University, Beijing, China; ^2^Department of Cardiology, Nanlou Division, Chinese PLA General Hospital, National Clinical Research Center for Geriatric Diseases, Beijing, China

## Abstract

**Background:**

Melatonin has been demonstrated to protect against calcification in cyclosporine nephrotoxicity. The wingless-type MMTV integration site family member 1 (Wnt1)/*β*-catenin pathway is associated with cardiovascular calcification. This study aimed to explore whether melatonin could attenuate VSMC calcification through regulating the Wnt1/*β*-catenin signaling pathway.

**Methods:**

The effects of melatonin on vascular calcification were investigated in vascular smooth muscle cells (VSMCs). Calcium deposits were visualized by Alizarin Red Staining. Calcium content and alkaline phosphatase (ALP) activity were used to evaluate osteogenic differentiation. Western blots were used to measure the expression of runt-related transcription factor 2 (Runx2), *α*-smooth muscle actin (*α*-SMA), and cleaved caspase-3.

**Results:**

Melatonin markedly ameliorated calcium deposition and ALP activity. Runx2 and cleaved caspase-3 were found to be reduced and *α*-SMA was found to be increased by melatonin, together with a decrease in apoptosis. Immunofluorescence assay revealed a lower Runx2 protein level in the melatonin group. Melatonin treatment significantly decreased the expression of Wnt1 and *β*-catenin. Treatment with lithium chloride or transglutaminase 2 abrogated the protective effects of melatonin.

**Conclusion:**

Melatonin can attenuate *β*-GP-induced VSMC calcification through the suppression of Wnt1/*β*-catenin system.

## 1. Introduction

Vascular calcification (VC) is prevalent in coronary artery disease, and the extent of VC predicts cardiovascular risk [1]. The causes of calcification in atherosclerosis include dysregulated matrix metabolism, epitaxial mineral deposition, inflammation, oxidative stress, and apoptosis [2]. VC mainly occurs in vascular smooth muscle cells (VSMCs) [3]. The wingless-type MMTV integration site family member 1 (Wnt1) protein plays important roles in the proliferation, differentiation, and death in many cells, and the Wnt1/*β*-catenin pathway is associated with cardiovascular calcification [4]. Melatonin is the main indoleamine produced by the pineal gland; it is known recently to have anti-inflammatory, anticancer, and antioxidant activities [5]. Several studies have shown that melatonin protects against VSMC inflammation and apoptosis [6, 7]. Melatonin also inhibits oxidative stress-induced apoptosis and calcification in endplate chondrocytes [8]. The aim of our study was to examine whether melatonin could attenuate VSMC calcification through regulating the Wnt1/*β*-catenin signaling pathway.

## 2. Materials and Methods

### 2.1. VSMCs Isolation, Culture, and Calcification

VSMCs were isolated from the aortas of Sprague–Dawley rats (4 weeks) using the explant method described in the previous study [9]. For calcification, VSMCs were cultured with Dulbecco's Modified Eagle Medium containing 10% fetal bovine serum and 10 mM *β*-glycerophosphate (*β*-GP) for 14 days [10]. For drug treatment, cultured VSMCs were incubated with melatonin before inducing calcification. And the melatonin receptor antagonist, luzindole (2 *μ*M), was added before inducing calcification [11]. To investigate the effects of melatonin on the Wnt1/*β*-catenin pathway, VSMCs were treated with lithium chloride (LiCl, the Wnt signaling agonist) (5 mM, Amresco, USA) [12] or transglutaminase 2 (TG2, the *β*-catenin signaling agonist) (0.0075 U/mL, Sigma, USA) [13] before inducing calcification.

### 2.2. Measurement of Calcium Deposition and Alkaline Phosphatase Activity

Alizarin Red S was performed to visualize the formation of mineralized matrix (Shanghai Gefan Biological Technology Co., Ltd, Shanghai, China). The calcium content was determined using a calcium colorimetric assay kit (Nanjing Jiancheng Biological Engineering Institute, Nanjing, China). Alkaline phosphatase (ALP) activity was measured using an ALP kit (Beyotime Institute of Biotechnology, Shanghai, China). Caspase-3 activity was analyzed using a caspase-3 assay kit (Beyotime Institute of Biotechnology, Shanghai, China).

### 2.3. Western Blots

VSMCs were lysed in RIPA buffer (Beyotime, China) for 30 minutes and then centrifuged at 14,000 ×g for 10 minutes at 4°C. Equal amounts of extracted protein samples were separated by sodium dodecyl sulfate-polyacrylamide gel and electrotransferred onto a polyvinylidene difluoride membrane (Millipore, MA, USA). Membranes were blocked with 5% nonfat dry milk in Tris-buffered saline containing 0.05% Tween 20 for 1 hour at room temperature followed by overnight incubation at 4°C with the following primary antibodies: anti-*α*-smooth muscle actin (*α*-SMA, 1 : 1000, Abcam, #ab32575), anti-runt-related transcription factor 2 (Runx2, 1 : 1000, Abcam, #ab76956), anti-cleaved caspase-3 (1 : 1000, Abcam, #ab13847), anti-Wnt1 (1 : 1000, Abcam, #ab15251), anti-*β*-catenin (1 : 1000, Abcam, #ab32572), and anti-*β*-actin (1 : 1000, Abcam, #ab8227). After overnight incubation, the membranes were further incubated with the appropriate secondary antibodies at room temperature for 60 minutes. Membranes were detected with an enhanced chemiluminescence reagent.

### 2.4. Quantitative Real-Time Polymerase Chain Reaction (qRT-PCR)

The one-step RT-PCR kit (TransGen Biotech Co., Ltd., China) was used to assess the reverse transcription of total RNA. The primers used for polymerase chain reaction were as follows: *α*-SMA, forward—5′-AAGTATCCGATAGAACAC-3′, reverse—5′-AAACATAATCTGGGTCAT-3′; Runx2, forward—5′-AGGCGCATTTCAGATGATGAC-3′, reverse—5′-ACCTGCCTGGCTCTTCTTAC-3′; *β*-actin, forward—5′-GATGGTGGGTATGGGTCAGAAGGAC-3′, reverse—5′-GCTCATTGCCGATAGTGATGACT-3′. The ABI PRISM 7500 Sequence Detection System using QuantiTect SYBR Green (QIAGEN, Hilden, Germany) was used to quantify differential gene expression. The mRNA expression levels were normalized to *β*-actin mRNA.

### 2.5. Immunofluorescence and TUNEL Method

For immunofluorescence, cells were fixed with 4% paraformaldehyde for 30 minutes, followed by permeation using 0.5% Triton X-100 for 10 minutes. Then, cells were blocked with 5% BSA for 1 hour and incubated with primary antibody against Runx2 (1 : 200, Cell Signaling Technology) and Wnt1 (1 : 200, Cell Signaling Technology) overnight at 4°C. The next day, cells were incubated with secondary antibody (1 : 200, Cell Signaling Technology) for 1 hour at 37°C. Apoptosis was detected using a TUNEL assay (Roche, Germany) according to the manufacturer's instructions.

### 2.6. Statistical Analysis

Data were expressed as the mean ± standard deviation (SD) of at least three independent experiments and were analyzed using one-way analysis of variance (ANOVA). The significance level was set at *P* < 0.05.

## 3. Results

### 3.1. Melatonin Inhibited *β*-GP-Induced VSMC Calcification

**Figure 1:**
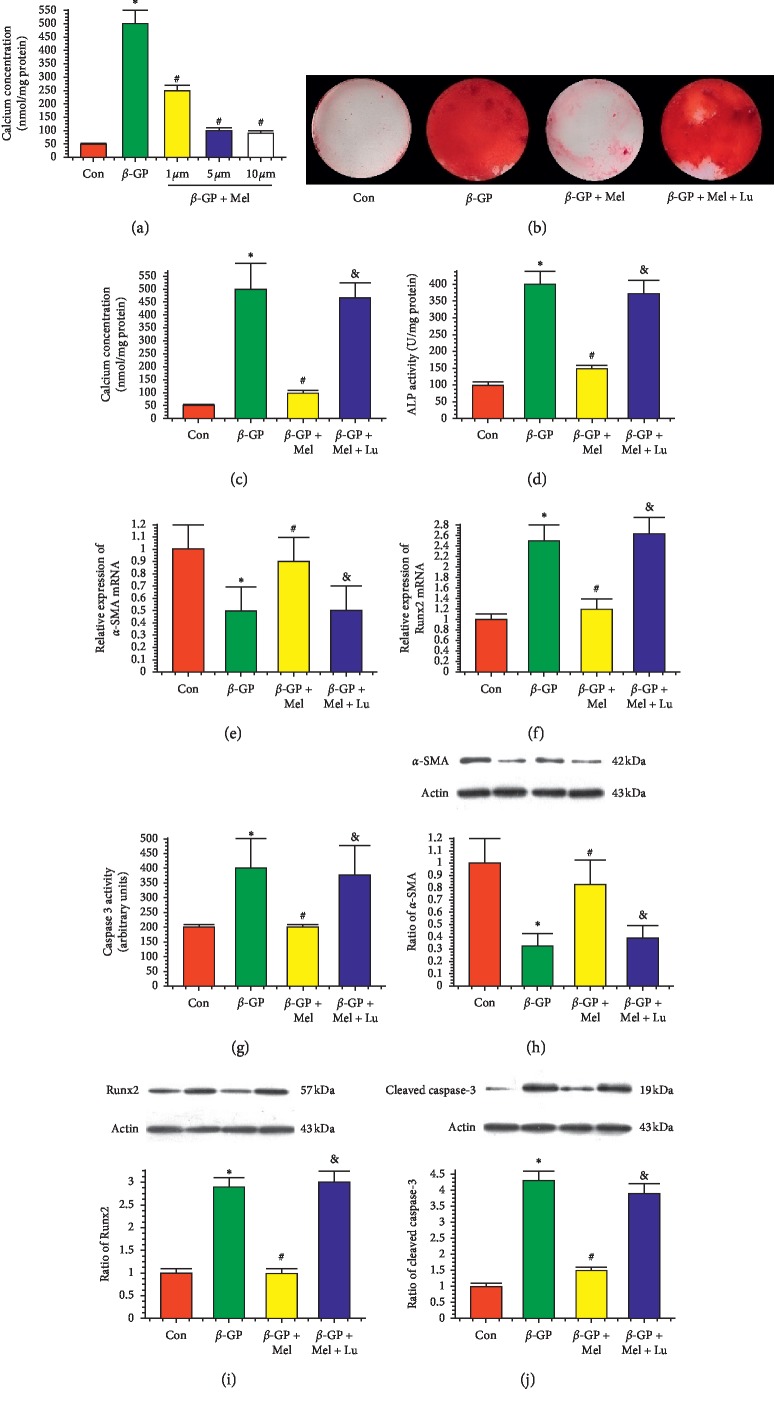
Melatonin reduced *β*-glycerophosphate-induced calcium deposition in vascular smooth muscle cells (VSMCs) (*n* = 3). VSMCs were cultured with Dulbecco's Modified Eagle Medium containing 10% fetal bovine serum and 10 mM *β*-glycerophosphate (*β*-GP) for 14 days. (a) Result of different concentrations of melatonin on calcium content. (b) Result of melatonin (5 *μ*M) on Alizarin Red Staining. (c) Result of calcium concentration. (d) Result of Alkaline phosphatase (ALP) level. (e–g) Results of *α*-smooth muscle actin (*α*-SMA) mRNA expression, runt-related transcription factor 2 (Runx2) mRNA expression, and caspase-3 activity. (h–j) Results of *α*-SMA, Runx2, and cleaved caspase-3 protein expression. ^*∗*^*P* < 0.05 versus Con, ^#^*P* < 0.05 versus *β*-GP, and &*P* < 0.05 versus *β*-GP + Mel.

As shown in Figure 1(a), 5 *μ*M of melatonin significantly reduced calcium content in calcifying VSMCs. Therefore, most experiments were performed at the concentration of 5 uM of melatonin. The Alizarin Red S indicated that *β*-GP promoted the calcification of VSMCs and melatonin significantly inhibited *β*-GP-induced calcification (*P* < 0.05) (Figures 1(b)–1(c)). ALP activity was significantly increased in response to *β*-GP, and melatonin significantly decreased ALP activity (Figure 1(d)). However, luzindole abrogated the protective effects of melatonin on VSMC calcification (*P* < 0.05). The mRNA expression of *α*-SMA was reduced in *β*-GP group as compared to the control group. The *β*-GP-induced reduction was mitigated by melatonin, but this effect was blocked by luzindole treatment. Moreover, the effects of melatonin on *α*-SMA protein expression in *β*-GP-treated VSMCs were consistent with the mRNA expression data. Runx2 mRNA and protein along with the ratio of cleaved caspase-3 were found to be upregulated in untreated cells, but in VSMCs treated with melatonin prior to *β*-GP, the levels were downregulated relative to the untreated cells (Figures 1(e)–1(j)).

**Figure 2:**
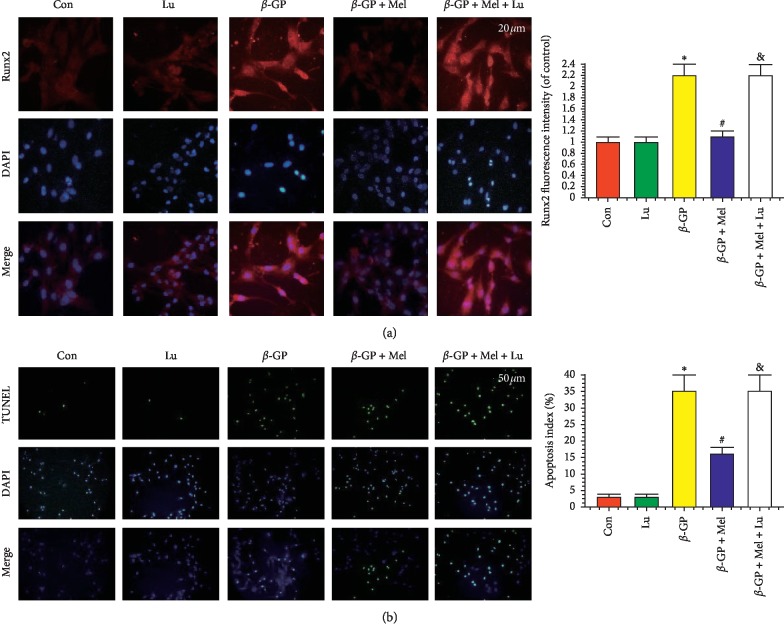
Effects of melatonin on the calcification and apoptosis in VSMCs (*n* = 3). (a) Confocal microscopy of immunofluorescence staining of Runx2 (red). (b) The apoptosis of VSMC was determined by TUNEL staining. ^*∗*^*P* < 0.05 versus Con, ^#^*P* < 0.05 versus *β*-GP, and &*P* < 0.05 versus *β*-GP + Mel.

Immunofluorescence assay was used to assess the expression of Runx2 protein in VSMCs. The Runx2 protein was increased in the *β*-GP group but decreased in the *β*-GP + melatonin group. But luzindole reversed this phenomenon (Figure 2(a)). Compared with the *β*-GP group, melatonin treatment significantly inhibited apoptosis in VSMCs (Figure 2(b)).

### 3.2. Melatonin Attenuated *β*-GP-Induced VSMC Calcification via Wnt1/*β*-Catenin Signaling

**Figure 3:**
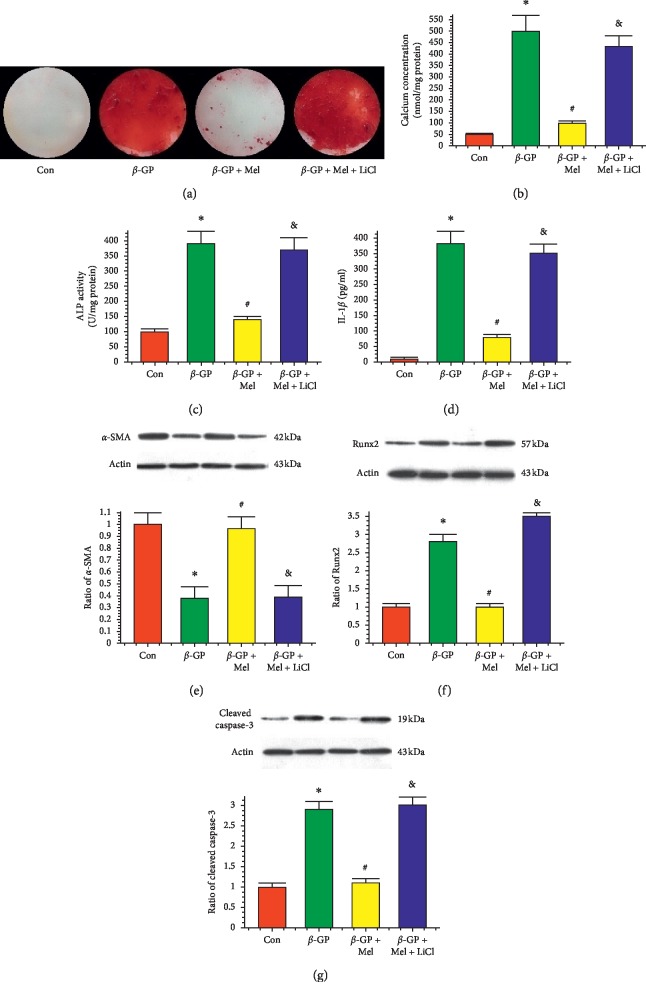
Effects of melatonin and lithium chloride (LiCl, 5 mM) on *β*-GP-induced calcium deposition in VSMCs (*n* = 3). (a) Result of Alizarin Red Staining. (b) Result of calcium concentration. (c) Result of ALP level. (d) Result of interleukin-1*β* (IL-1*β*) level. (e-g) Results of *α*-SMA, Runx2, and cleaved caspase-3 protein expression. ^*∗*^*P* < 0.05 versus Con, ^#^*P* < 0.05 versus *β*-GP, and &*P* < 0.05 versus *β*-GP + Mel.

Melatonin significantly reduced calcium deposition, ALP activity, and interleukin-1*β* level in *β*-GP-induced calcified VSMCs. But the Wnt1 activator LiCl reduced the protective effects of melatonin on VSMC calcification (Figures 3(a)–3(d)).

**Figure 4:**
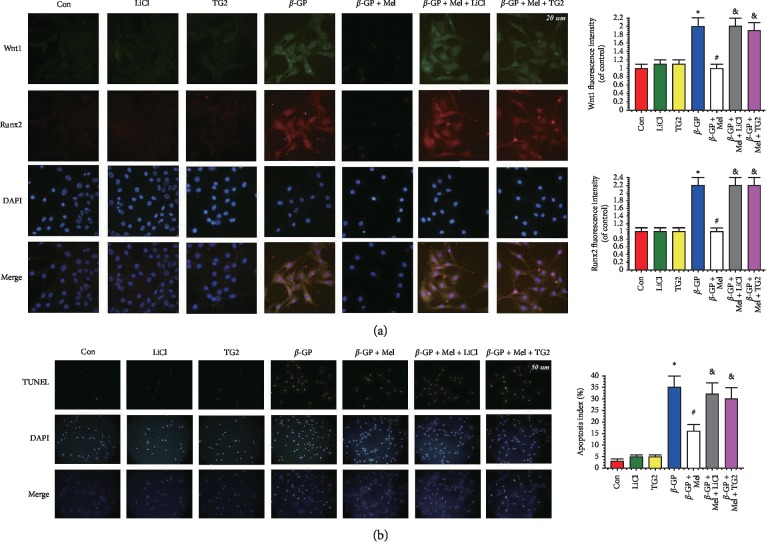
Effects of melatonin, lithium chloride (LiCl, 5 mM), and transglutaminase 2 (TG2, 0.0075 U/mL) on the calcification and apoptosis in VSMCs (*n* = 3). (a) Confocal microscopy of immunofluorescence staining of Wnt1 (green) and Runx2 (red). (b) The apoptosis of VSMC was determined by TUNEL staining. ^*∗*^*P* < 0.05 versus Con, ^#^*P* < 0.05 versus *β*-GP, and &*P* < 0.05 versus *β*-GP + Mel.

Melatonin significantly increased the protein level of *α*-SMA in calcifying VSMCs, but this melatonin-induced protection was nullified by LiCl. The expression levels of Runx2 and cleaved caspase-3 were increased in the *β*-GP group and were decreased in the *β*-GP and melatonin cotreatment group. However, LiCl increased these levels despite the treatment with melatonin (*P* < 0.05) (Figures 3(e)–3(g)). Immunofluorescence assay revealed a higher Wnt1 and Runx2 protein level in the *β*-GP + melatonin + LiCl and *β*-GP + melatonin + TG2 group (Figure 4(a)).

Melatonin significantly reduced the percentage of TUNEL-positive VSMCs compared with the *β*-GP group. The LiCl or TG2 ablated the protective effects of melatonin on apoptosis (Figure 4(b)).

### 3.3. Effect of Melatonin on Wnt1/*β*-Catenin Signaling

**Figure 5:**
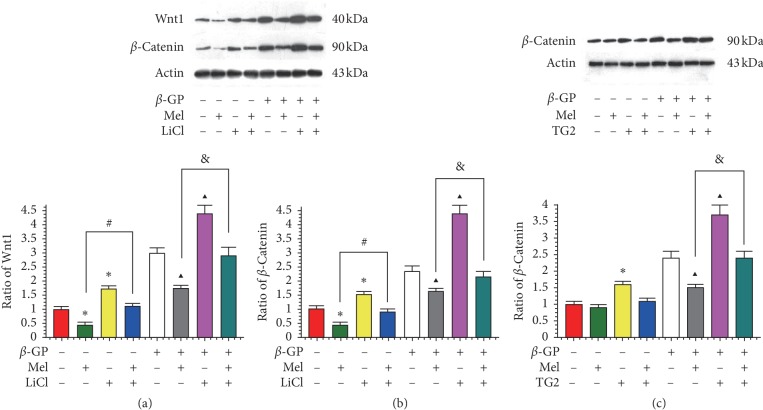
Effects of melatonin on the expression of Wnt1 and *β*-catenin in VSMCs (*n* = 3). (a-b) The effects of LiCl on the expression of Wnt1 and *β*-catenin. Cells were preincubated with or without LiCl (5 mM) stimulated with or without melatonin (5 *μ*M). (c) The effects of TG2 (0.0075 U/mL) on the expression of *β*-catenin. ^*∗*^*P* < 0.05 versus Con (without *β*-GP), ^#^*P* < 0.05 versus the values of cells incubated with melatonin, ^▲^*P* < 0.05 versus the values of cells incubated with *β*-GP, and &*P* < 0.05 versus the values of cells incubated with melatonin and *β*-GP.

Western blot was used to evaluate whether melatonin reduced the expression of Wnt1 and *β*-catenin in VSMCs. As shown in Figures 5(a) and 5(b), Wnt1 and *β*-catenin signaling was detected in VSMCs. Melatonin treatment significantly decreased the expression of Wnt1 and *β*-catenin. LiCl was administrated with and without melatonin treatment. A significant increase in Wnt1 was observed, as well as the suppression of the effects of melatonin on the expression of Wnt1 and *β*-catenin in the presence of *β*-GP-induced calcification (*P* < 0.05). We also found that TG2 alleviated the effects of melatonin on *β*-catenin (Figure 5(c)). These data demonstrate that melatonin represses the Wnt1/*β*-catenin signaling pathway in calcifying VSMCs.

## 4. Discussion

In the present study, we demonstrated the effects of melatonin on VSMC calcification, and the mechanism underlying these protective effects was revealed. Our results showed that the inhibition of VSMC calcification by melatonin is regulated, at least in part, through Wnt1/*β*-catenin signaling.

The effect of melatonin on calcification has been investigated more recently [8, 14, 15]. Son et al. found that melatonin could promote osteoblastic differentiation and mineralization of preosteoblastic MC3T3-E1 cells under hypoxic conditions [14]. But Kumar et al. showed that melatonin significantly antagonized cyclosporine-induced renal impairment. Microcalcification in corticomedullary junction seen with cyclosporine was inhibited by melatonin [15]. Zhang et al. demonstrated that melatonin treatment suppresses oxidative stress-induced apoptosis and calcification in endplate chondrocytes [8].

Wnt signaling plays a crucial role during embryogenesis. Recent studies have shown a reactivation of Wnt signaling in a variety of cardiovascular pathologies [16]. A recent study found that melatonin ameliorates estrogen deficiency-induced osteoporosis by suppressing the activation of the NLRP3 inflammasome via mediating the Wnt pathway [17]. Yu et al. reported that melatonin inhibits epithelial-mesenchymal transition in the lung alveolar epithelial cells, and the Wnt signaling pathway is involved in the epithelial-mesenchymal transition of the lung alveolar epithelial cells as they were suppressed by melatonin [18]. Similarly, our experiments confirmed that melatonin decreased the expression of Wnt1 and inhibited VSMC calcification. Furthermore, LiCl was used to increase the expression of Wnt1. We found that LiCl blocked the effects of melatonin on Wnt1 and aggravated VSMC calcification. Our data reveal that melatonin attenuates VSMC calcification through Wnt1 inhibition.

Wnt signaling pathways interact with glycogen synthase kinase 3 beta (GSK-3*β*) and *β*-catenin, promote Wnt target gene expression, and regulate many physiological processes. *β*-GP increased the expressions of Wnt, p-GSK-3*β,* and *β*-catenin, but they were reduced by pioglitazone treatment in rat vascular smooth muscle cells [19]. Yu et al. found that melatonin reduced endoplasmic reticulum stress during myocardial ischemia-reperfusion injury by enhancing the phosphorylation of GSK-3*β* [20]. Park et al. demonstrated that melatonin stimulated Wnt 5*α*/*β* protein expression and promoted the nuclear localization of *β*-catenin, while it inhibited GSK-3*β* phosphorylation in osteoblastic differentiation [21].

**Figure 6:**
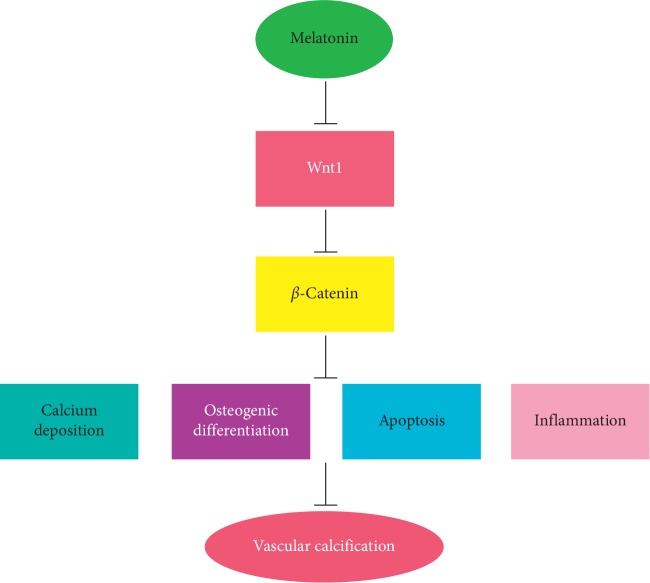
Schematic representation showing that melatonin regulates VSMC osteogenic differentiation through a Wnt1/*β*-catenin signaling pathway. Melatonin decreased the expression of Wnt1, which in turn inactivated *β*-catenin and subsequently suppressed osteogenic differentiation. These effects subsequently attenuated VSMC calcification.

A study by Shen et al. showed that melatonin inhibits neural cell apoptosis and promotes locomotor recovery via the activation of the *β*-catenin signaling pathway after spinal cord injury [22]. But the findings of Rhee and Ahn suggested that melatonin blocked the activation of peroxisome proliferator-activated receptor gamma which induced the degradation of *β*-catenin in human mesenchymal stem cells. Melatonin also decreased the levels of cyclic adenosine-3,5-monophosphate and reactive oxygen species [23]. Our data show that melatonin inactivated *β*-catenin. The Wnt1/*β*-catenin pathway activators, LiCl and TG2, decreased the effects of melatonin on *β*-catenin and increased VSMC calcification. Taken together, melatonin decreased the expression of Wnt1, which in turn inactivated *β*-catenin and subsequently suppressed osteogenic differentiation. These effects subsequently attenuated VSMC calcification (Figure 6).

There are a few limitations to our study. First, the findings are only based on in vitro experiments. Second, the siRNAs could be used to knock down Wnt1 or *β*-catenin to further validate our findings.

In conclusion, our study indicated that melatonin can inhibit *β*-GP-induced VSMC calcification through the suppression of Wnt1/*β*-catenin system.

## Data Availability

The authors declared that the data used to support the findings of this study are available from the corresponding author upon request.
